# Subcritical Water Extraction of Rosmarinic Acid from Lemon Balm (*Melissa officinalis* L.) and Its Effect on Plant Cell Wall Constituents

**DOI:** 10.3390/antiox12040888

**Published:** 2023-04-05

**Authors:** Ana Atanasova, Ani Petrova, Desislava Teneva, Manol Ognyanov, Yordan Georgiev, Nenko Nenov, Petko Denev

**Affiliations:** 1 Laboratory of Biologically Active Substances, Institute of Organic Chemistry with Centre of Phytochemistry, Bulgarian Academy of Sciences, 139 Ruski Blvd., 4000 Plovdiv, Bulgaria; 2 Department of Physiology, Pathophysiology, Chemistry, and Biochemistry, Prof. Dr. Assen Zlatarov University, 1 Prof. Yakim Yakimov Blvd., 8010 Burgas, Bulgaria; 3 InnoSolv Ltd., 4 Skayler Str., 4000 Plovdiv, Bulgaria

**Keywords:** lemon balm (*Melissa officinalis*), subcritical water extraction, antioxidant activity, phytochemical analysis, nutraceuticals, polysaccharides, lignin, plant cell walls

## Abstract

Rosmarinic acid (RA), an ester of caffeic acid and 3,4-dihydroxyphenyllactic acid, is a potent radical scavenger, a chelator of prooxidant ions, and an inhibitor of lipid peroxidation. RA-containing extracts are widely used natural antioxidants in food products, and many herbal preparations and food supplements, containing RA, are marketed with claims of beneficial health effects. The current study investigated the effectiveness of subcritical water extraction (SWE) for the recovery of RA from lemon balm (*Melissa officinalis*), as a “green” alternative to conventional hydro-alcoholic extraction. Different durations (10 min and 20 min) and extraction temperatures (100 °C and 150 °C) were applied. Subcritical water applied at a temperature of 100 °C was equally efficient as 50% ethanol in extracting RA. However, the further elevation of temperature to 150 °C decreased RA content by up to 20% due to thermal degradation. The content of RA in dried extracts was between 2.36% and 5.55% and the higher temperature of SWE increased extract yield by up to 41%. The higher extraction yield resulted from the degradation of plant material by subcritical water as evidenced by the increased extraction and degradation of proteins, pectin, and cellulose. These results reveal that SWE is an efficient technology for the extraction of RA and other antioxidants from lemon balm at reduced extraction time and without the use of toxic organic solvents. Furthermore, by modification of SWE conditions, dry extracts with different purity and content of RA are obtained. These extracts could be used in the food industry as food antioxidants, or in the development of food supplements and functional foods.

## 1. Introduction

Rosmarinic acid (RA), an ester of caffeic acid and 3,4-dihydroxyphenyllactic acid, is a widely distributed secondary metabolite in the plant kingdom. Many plant species, used traditionally in folk medicine, contain RA. Among them are lemon balm (*Melissa officinalis*), rosemary (*Rosmarinus officinalis*), thyme (*Thymus* spp.), and many other plant species, particularly from the Lamiaceae family. The amount of RA in these plants varies significantly, depending on environmental and genetic factors [1,2], but rarely exceeds 1% of the dry weight (DW) [3].

Many studies reveal that RA is a potent scavenger of reactive oxygen species, stronger in comparison to synthetic antioxidants such as BHT and BHA [4]. In addition, it demonstrates a higher reducing capacity in comparison to vitamin C, and strong chelating properties of pro-oxidant ions [5]. Due to its antimicrobial properties and ability to inhibit lipid peroxidation, RA is thoroughly investigated for its potential use in the food industry as a natural alternative to synthetic antioxidants and preservatives [6]. Extracts, containing RA, have been used for the extension of bread shelf-life [7], improving the taste of some culinary products, stabilizing corn oil [8], and used as a natural preservative in nonalcoholic beverages [9], and cupcakes [10]. Furthermore, RA revealed a strong co-pigmentation effect towards black chokeberry anthocyanins, thus increasing their stability and color intensity [11]. Currently, the only RA-containing plant extract officially allowed as a food preservative in the European Union (EU) is rosemary extract (E392) [12]. However, recently EFSA assessed the safety of a dried extract (containing ≥ 10% of hydroxycinnamic acid derivatives including ≥ 3% of RA) from the leaves of *Melissa officinalis* L. and concluded that it could be used as a feed additive for all animal species.

RA exhibits numerous health benefits, related mainly to its neuroprotective [13], hepatoprotective [14], and anti-inflammatory properties [15,16]. For example, it inhibits certain enzymes related to diabetes [17], as well as white adipogenic differentiation, and induces adipocyte basal lipolysis in human adipocytes [18]. RA revealed hepatoprotective properties by reducing lipid peroxidation, degree of fibrosis, and overall improving biochemical parameters of the liver [19]. Due to the strong anti-inflammatory effect, the administration of RA-containing extracts significantly reduced pain in patients with osteoarthritis. In addition, it positively affected colitis and inflammatory skin conditions such as atypical dermatitis, in line with other inflammation-related conditions (e.g., asthma, allergic rhinitis) [15,16,20,21]. Furthermore, extracts containing RA revealed no detrimental side effects on the liver, kidneys, and other organs [22]. For that reason, a large number of RA-containing preparations and food supplements are marketed with health claims.

Lemon balm (*Melissa officinalis* L.) is among the medicinal plants rich in RA, with up to 6% RA in dry leaves [23]. Due to the abundance of biologically active substances such as hydroxycinnamic acid derivatives (i.e., caffeic and chlorogenic acids), essential oil (0.06–0.8%), flavonoids, tannins [24], and triterpenes [23], lemon balm has been used for centuries in folk medicine for different ailments. The most popular health benefits of lemon balm and its extracts are related to calming and anxiolytic effects and the majority of medicines and nutraceuticals based on lemon balm are intended to improve sleep quality or for treating insomnia [25,26]. Besides food supplements, standardized herbal substances or extracts from lemon balm leaves are recognized on the EU level as a traditional herbal medicinal product for the relief of mild symptoms of mental stress and to aid sleep and traditional herbal medicinal products for symptomatic treatment of mild gastrointestinal complaints, including bloating and flatulence [27]. However, for that purpose, European Pharmacopoeia requires a minimum content of 1% RA in dry lemon balm leaves (*Melissae folium*) used for herbal preparations [28] and a minimum of 2% RA in dry extracts produced from *Melissa* leaf [29].

Besides the biotechnological approaches of RA via in vitro systems [30], plants remain the most important and widely exploited source of RA. Some physical (i.e., controlled irradiation with light) [31] and chemical methods have been applied to increase its accumulation or recovery from plants. Conventional chemical methods commonly used for the isolation of RA from plants include Soxhlet extraction, maceration, heat reflux extraction, and soaking, and the most frequent solvents used are methanol, ethanol [32,33], or hydro-alcoholic solutions [34]. While the yield of RA is high, the extraction time is often too long [35,36,37]. To overcome that obstacle, newer techniques such as microwave and ultrasonic extraction have been used, as they are performed in a shorter time [38]. Enzyme-assisted extraction has also been used, where cellulase enzymes are used to disturb cell walls and enhance the extraction of RA and polyphenols [39].

In recent years, the concept of “green chemistry” has gained prominence, with a focus on finding extraction methods that protect the environment and are non-toxic to humans. Thus, several rapid, environmentally friendly, and clean extraction techniques for RA, such as supercritical carbon dioxide extraction [40], pressurized liquid extraction [41], ionic liquids-based extraction [42], and molecularly imprinted polymer selective extraction have been researched [43]. Subcritical water extraction (SWE) is a relatively new technique for extracting biologically active substances using water as an extractant under subcritical conditions (temperatures between 100–374 °C and pressures sufficient to maintain a liquid phase). SWE is also an environmentally friendly method as it does not use toxic organic solvents [44]. The main advantages of SWE are related to the use of water instead of organic solvents and the reduced time, which significantly reduces economic costs and the environmental footprint of the extraction process. So far, SWE has been used for the recovery of various bioactives from different plant matrices [45], but there is only evidence in the literature for the application of subcritical water for the extraction of lemon balm [8]. However, the study emphasizes the application of the extract for the stabilization of corn oil, without presenting detailed chemical characterization or assessing the efficiency of the extraction process [8]. To the best of our knowledge, comparing the extraction efficiency of subcritical water with conventional water or water-alcoholic extraction has not been performed. Therefore, the study aimed to investigate the extraction efficiency of RA and the major phenolic antioxidants from lemon balm under different subcritical conditions in comparison to conventional hydro-alcoholic extraction. In order to explain the better effectiveness of subcritical water for RA extraction, we also analyzed carbohydrates (uronic acids, cellulose, and free sugars), lignin, and protein contents in the obtained extraction residues, which are indicative of the degree of plant cell wall degradation. The obtained results are discussed in light of the available regulatory requirements, thus paving the way for the potential use of the extracts as nutraceuticals, traditional herbal medicinal products, and feed/food additives.

## 2. Materials and Methods

### 2.1. Plant Material

Lemon balm (*Melissa officinalis* L.) cut leaves (Batch L CS26.2/1209/22; net weight: 4 kg) were purchased from Botanical EU Ltd. (Cheshnegirovo, Plovdiv region, Bulgaria) in September 2022 and kept in paper bags. Dried leaves were ground to a fine powder immediately before extraction and analysis.

### 2.2. Proximate Composition Analysis of Plant Material

For the determination of moisture content, the milled sample (~1.3 g) was dried in an automated moisture analyzer (KERN DLB 160-3A, Bensheim, Germany) at 105 °C until constant weight. The ash content was determined as the pulverized sample (0.5 g) was placed in a crucible and ignited in a muffle furnace at 550 °C until there was no change in the mass of the sample. For the estimation of crude lipid content, the ground sample (10.0 g) was packed in a cellulose thimble and subjected to exhaustive extraction with petroleum ether (500 mL) for 8 h in a Soxhlet extractor. The obtained crude extract was dried under vacuum and its weight was used for the calculation of the lipid content. The crude protein content was evaluated by the micro-Kjeldahl method (N × 6.25). The determination of nitrogen expressed as ammonia content of the digested sample was performed using the acetylacetone–formaldehyde colorimetric method using ammonium sulfate as a standard [46]. The total carbohydrate content of the leaves was analyzed by the phenol-sulfuric acid method using glucose for the calibration curve construction [47]. The sample was solubilized in 72% (*w*/*w*) H_2_SO_4_ (1 h, 30 °C), and after dilution with water to 1 M H_2_SO_4_, hydrolysis was completed in 3 h at 100 °C. The obtained hydrolyzate was used as a sample for analysis. The absorbance was measured at 490 nm.

### 2.3. Extraction of RA and Phenolic Components

#### 2.3.1. Reference Extraction Procedure of RA and Phenolic Components

A pharmacopoeian method was used as a reference extraction procedure of phenolic components including RA [29]. Briefly, 0.1 g of the powdered plant material was weighed accurately and mixed with 90 mL 50% (*v*/*v*) ethanol in a brown-glass round-bottomed flask. The mixture was boiled in a water bath under a reflux condenser for 30 min, cooled, and filtered into a 100 mL volumetric flask. Flask and filter were rinsed with 10 mL of 50% ethanol and diluted to 100.0 mL with the same solvent. The extraction procedure was repeated two times and the extract was denoted as Control (C) extract.

#### 2.3.2. Static Maceration (Preparation of Tincture)

For this procedure, 4 g of the powdered plant material was mixed with 80 mL 50% (*v*/*v*) ethanol (solid-to-liquid ratio of 1:20) in brown-glass flasks. Flasks were left at room temperature for 30 days and shaken by hand every day. Periodically, 500 µL of the extracts were taken for analysis after centrifugation (6000× *g*, 20 min). The extraction procedure was repeated two times and the extract was denoted as Static Maceration (SM) extract. It was observed that the highest content of phenolic compounds including RA was extracted on the 10th day; therefore, all results denoted with SM refer specifically to this sample.

#### 2.3.3. Temperature-Assisted Dynamic Maceration

For this procedure, 4 g of the powdered plant material was mixed with 80 mL 50% (*v*/*v*) ethanol (solid-to-liquid ratio of 1:20) in 100 mL screwcap pyrex glass bottles. Bottles were shaken in a water bath (NUVE, Asagi Ovecler Ankara, Turkey) for 1 h, at 60 °C. The bottles were then cooled down and the extract was filtered. The extraction procedure was repeated two times and the extract was denoted as Temperature-Assisted Dynamic Maceration (TADM) extract.

#### 2.3.4. Subcritical Water Extraction

Static subcritical water extraction was performed on a 2 L capacity device, equipped with a vessel for electrical heating of water, heat-insulated extractor (useful volume 2.0 L), circulation pump for hot water, cooler, and electric control panel, as described in detail elsewhere [48]. Two different extraction temperatures (100 °C and 150 °C) and two different extraction times (10 min and 20 min) were employed for the extraction of RA and a counterpressure of 10 bar was applied to keep water liquid by pumping nitrogen into the system. For the extraction, 100 g of plant material was loaded into a cylindrical metal sieve and put into the extractor. The system was filled with 2 L (solid-to-liquid ratio of 1:20) water, which after reaching the desired temperature was pumped continuously (for the desired time) through the metal sieve loaded with plant material. After that, extracts were cooled down to room temperature directly in the equipment. All extractions (for each temperature and duration) were repeated twice and the extracts denoted SWE100/10, SWE100/20, SWE150/10, and SWE150/20, reflecting extraction temperature and duration.

### 2.4. Total Polyphenol Content

Total polyphenols were determined according to the method of Singleton & Rossi (1965) [49] with the Folin-Ciocalteu’s reagent. Gallic acid was employed as a calibration standard and the results were expressed in mg Gallic acid equivalents (GAE) per 100 g DW ± SD (*n* = 8).

### 2.5. Antioxidant Activity Assays

Oxygen radical absorbance capacity (ORAC) activity was measured on a microplate reader FLUOstar OPTIMA (BMG Labtech, Ortenberg, Germany) with excitation at λ = 485 nm and emission at λ = 520 nm, according to the method of Ou et al. [50] with some modifications by Denev et al. [51]. Trolox was used for building the standard curve and results were expressed in micromole Trolox equivalents (μmol TE) per gram DW ± SD (*n* = 8).

Hydroxyl radical averting capacity (HORAC) activity was determined with excitation at λ = 485 nm and emission at λ = 520 nm, according to Ou et al. [52]. Gallic acid was used for the standard curve and results were expressed in micromole gallic acid equivalents (μmol GAE) per gram DW ± SD (*n* = 8).

### 2.6. High Performance Liquid Chromatography (HPLC) Determination of RA

RA content in the dry herb and dried extracts was analyzed and calculated according to the Ph Eur monographs 1447 and 2524 [28,29], respectively with a slight modification in the chromatographic conditions. A HPLC system Nexera-i LC2040C Plus (Shimadzu Corporation, Kyoto, Japan) with a UV-VIS detector and a binary pump was used. The column was Poroshell 120 EC-C18 (3 mm × 100 mm, 2.7 μm), thermostated at 26 °C. The flow rate was 0.3 mL/min and the injection volume was 5 μL. The detection of the derivatives was made at λ = 330 nm. The mobile phase consisted of A: phosphoric acid, acetonitrile, water (1:19:80 *v*/*v*/*v*) and B: phosphoric acid, methanol, acetonitrile (1:40:59 *v*/*v*/*v*). The gradient started with 100% A and changed gradually to 55% A and 45% B at 20 min. After that, between 20 min and 25 min B reached gradually 100%. From 25 min to 30 min A changed from 0% to 100%. 

The content of RA (%) in herb/extract was calculated using the following expression:

$$\frac{A1\times M2\times P\times 0.2}{A2\times M1}$$

where:

*A*1 = area of the peak due to RA in the chromatogram obtained with the test solution;
*A*2 = area of the peak due to RA in the chromatogram obtained with reference solution;
*M*1 = mass of the plant material/extract to be examined used to prepare the test solution, in grams;
*M*2 = mass of RA used to prepare reference solution, in grams;
*P* = percentage content of RA in standard rosmarinic acid.

### 2.7. HPLC Determination of Other Phenolic Compounds

HPLC analyses were performed on an UHPLC system Nexera-i LC2040C Plus (Shimadzu Corporation, Kyoto, Japan) with a UV-VIS detector and a binary pump. The column was Poroshell 120 EC-C18 (3 mm × 100 mm, 2.7 μm), thermostated at 26 °C. The flow rate was 0.3 mL/min and the injection volume was 5 μL. The derivatives were detected at λ = 280 nm. The mobile phase consisted of A: 0.5% acetic acid and B: 100% acetonitrile. The gradient condition started with 14% (B), between 6 and 30 min, linearly increased to 25% (B), and then to 50% (B) at 40 min. The identification of compounds was confirmed by a comparison of retention times utilizing standard solutions and standard calibration curves of different phenolics (gallic acid, neochlorogenic acid, 3,4-dihydroxybenzoic acid, chlorogenic acid, catechin, vanillic acid, caffeic acid, epicatechin, *p*-coumaric acid, ferulic acid, rutin, ellagic acid, quercetin-3-*β*-glucoside, naringin, rosmarinic acid, myricetin, cinnamic acid, quercetin, luteolin, naringenin, apigenin, and kaempferol). The results for individual phenolic compounds were expressed in mg per 100 g DW ± SD.

### 2.8. HPLC Determination of Free Sugars

One gram of the powdered plant material was extracted (1 h, 30 °C) with *meta*-phosphoric acid (30 mL, 3%) using continuous shaking in a water bath. The extract was recovered through centrifugation (6000× *g*, 20 min) and further filtration through a PTFE filter (0.45 μm). The filtrated extract was used for chromatographic analysis. The analysis of free sugars was performed on a ZORBAX Carbohydrate (5 μm, 4.6 × 150 mm, Agilent) and a ZORBAX Reliance Cartridge guard column. An UHPLC system Nexera-i LC2040C Plus (Shimadzu Corporation, Kyoto, Japan) with a binary pump and a 20 A refractive index detector. The sample was eluted at a flow rate of 1.0 mL/min at 25 °C with a mobile phase composed of a mixture of acetonitrile and water (80:20 *v*/*v*). The concentration of sugars, detected by their retention time, in the sample was obtained using a calibration curve built by plotting the peak area against the concentrations of each standard.

### 2.9. Uronic Acid, Cellulose, and Lignin Content

For the estimation of the uronic acid content of the raw material and polysaccharides (PSs) an automated 3-hydroxybiphenyl method was conducted by a Skalar San^++^ apparatus (Skalar Analytical BV, Breda, the Netherlands), following the manufacturer’s instructions. Absorption was measured at 530 nm and galacturonic acid (12.5–100.0 μg/mL) was used as a standard. Preliminary, the sample was threefold-extracted with ethanol (70% (*v*/*v*), 50 °C, 1 h). The mixture was centrifugated (18.187× *g*) to separate solid material before each repetition. In addition, the residue was soaked (2×, 1 h) with pure acetone at room temperature and vacuum dried. Finally, the sample was hydrolyzed as described in Section 2.2. and the filtrated hydrolyzate was run as a sample for analysis. PSs samples were directly analyzed by the method after proper dissolution and dilution.

The quantitative estimation of cellulose was performed spectrophotometrically according to the semi-micro method of Updegraff with a modification [53]. Briefly, a sample (30–35 mg) was gently boiled (30 min) with 2 mL of acetic acid-HNO_3_ reagent (acetic acid:H_2_O:HNO_3_ 8:2:1 *v*/*v*/*v*) in a microtube with O-ring screw cap. After cooling the insoluble residue was separated through centrifugation, and then washed with deionized water to neutral pH. The obtained residue was subjected to sulfuric acid (72% *w*/*w*) treatment to solubilize cellulose and which was further determined by the phenol-sulfuric acid method after an appropriate dilution.

A color reaction with phloroglucinol-hydrochloric acid (Wiesner’s test) was conducted as a procedure for the detection of lignin. The lignin content was evaluated by the Klason lignin gravimetric method (KL) [54]. Prior to performing the color reaction and analysis, the plant material was successively extracted in a Soxhlet apparatus with water, ethanol (96% *v*/*v*), chloroform:methanol (2:1 *v*/*v*), and acetone to obtain a cell wall material [55].

### 2.10. Isolation of the Polysaccharide Fractions

A volume of 500 mL of the obtained extracts (M1-4) was used for the isolation of PS fractions. The extracts were initially centrifuged (20 min, 5000× *g*). The volume of the supernatant was reduced in half. Then, the supernatant and 96% (*v*/*v*) cold ethanol were combined in a ratio of 1:2 to precipitate PSs. The mixture was centrifuged (20 min, 4 °C, 3490× *g*) to recover the precipitate. It was then re-dissolved in distilled water and extensively dialyzed (mwco 3.5 kDa, Visking^®^, SERVA Electrophoresis, Heidelberg, Germany) for 72 h against distilled water (4 °C). Finally, the dialysate was frozen in plastic containers (100 mL), which were freeze-dried in an Alpha 1–4 LDplus laboratory freeze dryer (Martin Christ Gefriertrocknungsanlagen GmbH, Osterode am Harz, Germany).

### 2.11. Preparation of Freeze-Dried Extracts

For the preparation of freeze-dried extracts, ethanol from TADM was rotary evaporated at 50 °C under vacuum, whereas subcritical water extracts were only filtered. After that, 100 mL from each extract were poured into plastic containers, frozen and freeze-dried in an Alpha 1–4 LDplus laboratory freeze dryer (Martin Christ Gefriertrocknungsanlagen GmbH, Osterode am Harz, Germany).

### 2.12. Statistical Analysis

All extractions were performed in duplicate. The HPLC analyses were performed twice for every single sample (*n* = 4), whereas other analyses were run at least in triplicate for each sample (*n* = 6). Results were expressed as mean values ± standard deviations. One-way analysis of variance (ANOVA) and Student’s *t*-test were used to evaluate the differences in the mean between groups. *p* values less than 0.05 were considered to be significant. Microsoft Excel, 2013 (Microsoft Corporation, Redmond, WA, USA) was used in the analyses.

## 3. Results and Discussion

### 3.1. Proximate Composition Analysis of Lemon Balm Leaves

As the first step of the study, we analyzed the proximate chemical composition of lemon balm, which is very informative when assessing the effect of subcritical water on plant matter and its efficiency in extracting RA (Table 1). The table shows that carbohydrates were the major component of lemon balm (22% DW), followed by proteins and lignin. Lipid components were found in significantly smaller quantities. Cellulose and pectin (uronic acids) represented approximately 50% of the total carbohydrates. As could be seen, the raw material was a very rich source of phenolic compounds −11.5%, including 1.60% RA. From the investigated 22 phenolic compounds, besides RA, we also identified and quantified caffeic acid, neochlorogenic acid, and luteolin. However, they were presented in very low amounts, confirming that RA is the major biologically active component in lemon balm leaves. Chromatograms from two analyses are presented in Supplementary Figure S1. The high content of phenolic compounds rendered a relatively high antioxidant activity measured by ORAC (1814.6 ± 51.9 µmol TE/g DW) and HORAC (950.2 ± 15.9 µmol GAE/g DW) methods, respectively. The raw material was very rich in crude protein and contained substantial amounts of minerals, expressed as 18.1% ash.

### 3.2. Recovery of Rosmarinic Acid from Lemon Balm Leaves by Subcritical Water and Conventional Hydro-Alcoholic Extraction

**Figure 1:**
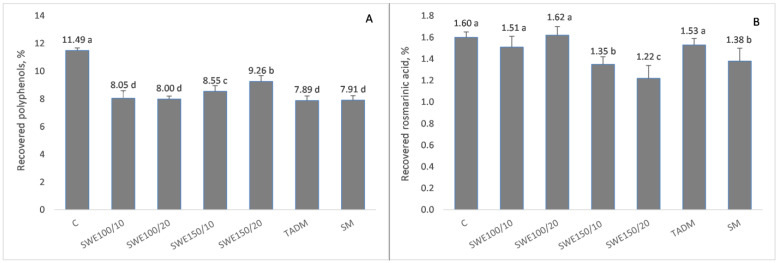
Effect of extraction method on the recovery of total polyphenols (panel (**A**)) and rosmarinic acid (panel (**B**)) from lemon balm leaves. Results are presented as mean values ± SD. There are no significant differences among values marked with the same letters (a, b, c, d) within individual groups.

There are many examples from the literature supporting that methanol, ethanol, and hydro-alcoholic solutions are better extragents of RA in comparison to water [56,57]. However, it should be noted that with an increase in temperature below the critical point, the dielectric constant of water decreases, and favors the dissolution of substances that are otherwise more soluble in organic solvents. Therefore, we hypothesized that under subcritical conditions, water could be an efficient solvent of RA from lemon balm. To assess the applicability of SWE for the extraction of RA, without the use of organic solvents, two different extraction temperatures (100 °C and 150 °C) and durations (10 min and 20 min) were tested. The efficiency of SWE for recovery of RA from lemon balm dry leaves was compared with two commonly used methods for hydro-alcoholic extraction–SM and TADM. In order to have an objective positive control, all results were compared to those of the pharmacopoeian method for the extraction of RA [28]. Since this method employs 50% (*v*/*v*) ethanol, SM and TADM were also performed with 50% ethanol and both SWE and hydro-alcoholic extractions were carried out with a plant material-to-extragent ratio of 1:20, thus allowing easier and more objective comparison of the results. As a preliminary step, we investigated the recovery of phenolic compounds using static maceration for a period of 30 days and observed that the highest contents of phenolic compounds and RA were extracted on the 10th day (results are presented in Supplementary Figure S2). Therefore, results denoted with SM refer specifically to that sample. Figure 1 presents data for the recovery of RA and total phenolic compounds from lemon balm leaves depending on the extraction method used. All results are calculated and presented on the basis of dry herb.

Subcritical water was an efficient solvent of RA from *Melissa officinalis* leaves. The referent pharmacopoeian method used for extraction provided a very exhaustive extraction of total phenolic compounds (11,491 mg/100 g DW), which exceeded significantly (*p* < 0.05) the yield of total phenolics extracted by other techniques. However, this was not the case with RA (Figure 1, panel B). Practically, subcritical water extraction at 100 °C, regardless of the extraction time, was equally efficient in the recovery of RA from the plant material compared to the pharmacopoeian method and TADM with 50% ethanol. Increasing the temperature to 150 °C decreased significantly (*p* < 0.05) the amount of extracted RA, which is most probably due to its thermal degradation. It is known that rosemary extract constituents, including RA, degraded because of increased temperature and exposure to light [58] or gamma-irradiation [59]. The amount of RA yielded by static maceration, which is the classical method for tincture preparation, was significantly lower in comparison to TADM and SWE at 100 °C. It should be noted that results for RA recovery were obtained from the 10th day of maceration when the maximum amount of total polyphenols was yielded. As could be seen from Supplementary Figure S2, after the 10th day, the amount of extracted RA decreased significantly, which is further evidence of its labile nature. It is known that polyphenols, including RA, are prone to oxidation into quinones, which further can react with nucleophilic group thiols and amines, leading to the formation of different adducts with amino acids and proteins [60,61]. Regardless of the decreased RA content, water at a higher temperature (150 °C) extracted more polyphenols in comparison to SWE at 100 °C and hydro-alcoholic extractions. This could be explained by the increased degradation of plant cell walls and liberation of additional polyphenols from the degraded plant matter, which is demonstrated and discussed in detail in Section 3.4.

### 3.3. Characterization of Lemon Balm Dried Extracts, Obtained by Subcritical Water or Hydro-Alcoholic Extraction

**Figure 2:**
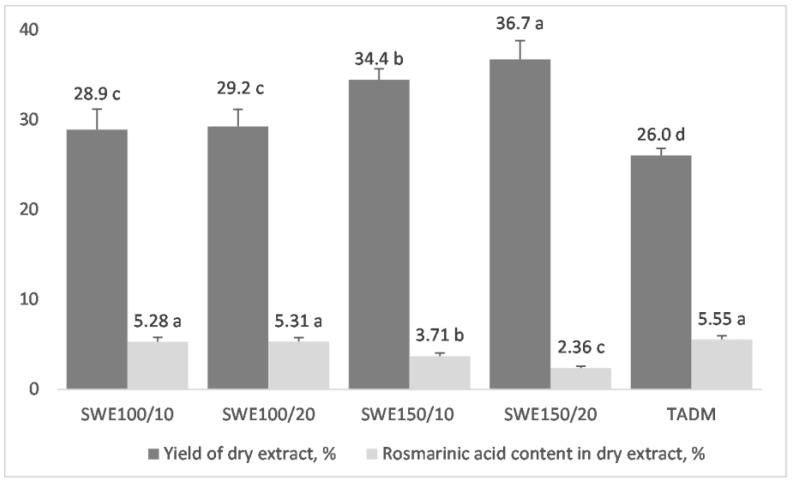
Yield of extract and rosmarinic acid content of dry extracts, obtained by different extraction methods. Results are presented as mean values ± SD. There are no significant differences among values marked with the same letters (a, b, c, d) within individual groups.

As it was demonstrated in Section 3.2, SWE is equally efficient as TADM and superior to SM in extracting RA from lemon balm leaves. However, dried standardized extracts are the most widely used form for the application of medicinal plants in the food and nutraceutical industries. Therefore, subcritical water extracts were freeze-dried and analyzed. The results were compared to the TADM extract, which was freeze-dried after the removal of ethanol. As could be seen in Figure 2, the yield of dry extract varied significantly from 2.36% to 5.55%. It should be noted that in all cases, extracts contained more than 2% RA, which is required by European pharmacopoeia for the *Melissa officinalis* dried extract [29]. The highest content of RA was found in TADM and subcritical extracts obtained at 100 °C for both 10 min and 20 min (*p* < 0.05).

Subcritical water gave a higher extraction yield than TADM. Even at the lower temperature, the extract yield was 11.5% (*p* < 0.05) higher in comparison with the yield of hydro-alcoholic extraction. The duration had no significant effect (*p* < 0.05) on the yield of extract, even though there was a trend towards increasing. On the other hand, the extraction temperature had a profound effect and SWE150/20 yielded 41% more extract in comparison to TADM. However, the higher extraction yield and degradation of RA at the elevated temperature decreased the purity of RA in the extract to 2.36%. The higher extraction yield is most probably due to the better extraction of pectic PSs and proteins, and partial degradation of cellulose, resulting from the high temperature, which will be demonstrated and discussed in Section 3.4.

In all cases, RA was the major phenolic compound and it represented approximately 90% of the phenolics in extracts, obtained with subcritical water at 100 °C and with 50% ethanol. Besides RA, caffeic acid, neochlorogenic acid, and luteolin were quantified in the extracts (Table 2). Hot water was a better extragent for caffeic and neochlorogenic acid than 50% ethanol. Luteolin and its glycosides are the major flavonoids found in lemon balm [62,63], and it is very interesting that as a less polar compound, luteolin was extracted with the same efficiency by 50% ethanol and subcritical water. Further increases in water temperature to 150 °C increased several-fold the amount of extracted luteolin, which could be associated again with the degree of degradation of plant material, but also with the hydrolysis of luteolin glycosides and liberation of the aglycon. Interestingly, increasing the hot water temperature from 100 °C to 150 °C doubled the amount of free caffeic acid in the extract, which is probably due to the hydrolysis of caffeic acid dimers, trimers, and tetramers, previously identified in lemon balm [63]. Furthermore, we assessed the antioxidant properties of the obtained dry extracts by two methods–ORAC and HORAC. In general, total polyphenol content correlated well with antioxidant activity. However, samples with the highest polyphenol content (subcritical extracts obtained at 100 °C) revealed lower peroxyl radical scavenging (ORAC) and hydroxyl radical averting capacity (HORAC) values than SWE150/20, indicating that antioxidant properties of the investigated extracts were strongly influenced by the individual phenolic profile of each extract.

### 3.4. Influence of Subcritical Water Extraction on Primary and Secondary Cell Wall Constituents

As has already been discussed, subcritical water is a suitable extragent for RA from lemon balm and a higher extraction yield was achieved with subcritical water in comparison to ethanol. Furthermore, the higher water temperature increased the yield of extract. To explain these observations and to assess better the effect of water under subcritical conditions on the plant material, we analyzed the protein, cellulose, total uronic acid, and lignin contents of the residues after extraction. The results are given in Table 3. As it is evident from the results, both extraction time and extraction temperature affect the components of the primary cell walls.

The protein content of the residues after extraction did not change (21–22%). Given the residue yield after extraction, it is clear that 56% of the total amount of crude protein found in the initial material was recovered in the SWE150/20 residue. Under the softer extraction conditions (SWE100/101), this percentage was much higher (84%), which suggests that a significant portion of the initial proteins are still present in the residue, and only 16% has passed into the extract. The tendency in the uronic acids, which reflect the content of acidic PSs (e.g., pectin), was similar. Their content in residues after SWE150/10 and SWE150/20 decreased by 2–3 times, indicating that some of them were extracted and passed into the respective extract. Particularly interesting is the fact that only 28% of the initial uronic acids were recovered in the residue after SWE150/20 extraction, which indicates that 72% of them have passed into a soluble form and were extracted from the raw material. Under softer extraction conditions, it turns out that the cell walls were not affected, because residues after extraction contain high amounts of the initial uronic acids. The situation was similar to that of cellulose, which is the main building skeleton of plant cell walls. However, there were no major changes in cellulose induced by SWE. It turns out that it was much more resistant to the conditions of extraction than uronic acids. In the residues after extraction, there were between 105% and 91% of the initial cellulose, suggesting that this polysaccharide was poorly affected. Only at the higher temperature and longer extraction duration began the destruction of the cellulose skeleton. In the residue after SWE150/20 extraction, only 79% of the initial cellulose was recovered. As a result of subcritical extraction, the content of the acid-insoluble lignin increased in the residues, reaching nearly 41% in SWE150/20 residue. It is especially interesting that, unlike polysaccharide components that undergo destruction to a certain extent, lignin did not seem to change as a result of subcritical water treatment. This was also evident from the analytical recovery of lignin in the residues after extraction, which contain between 99% and 104% of the initial lignin present in lemon balm leaves (Table 1 and Table 3).

Table 4 presents data for the PS content and free sugars of the extracts, as well as the total carbohydrate and uronic acid contents of isolated PSs. The total carbohydrate content of the PSs varied between 31% (SWE100/10) and 66% (SWE150/10). The yield of PS nearly doubled with an increase in the extraction duration and temperature. In addition, while the carbohydrate content of PSs increased, that of the uronic acids decreased by 50%. Some oligosaccharides (cellodextrins, etc.) released from water-insoluble or strongly cross-linked polymers, such as cellulose, could be expected, as illustrated by the data in Table 3. The SWE extracts contained representative amounts of uronic acids, which were suggested to derive from pectic PSs. Therefore, a significant amount of the PSs in the samples was of a pectic nature. It is obvious that more severe thermal-assisted decompositions occurred during the extraction at 150 °C than at 100 °C. This can be also clearly seen from the recovery data (Table 4). Judging by the content of uronic acids, it turns out that PSs contained a very small part of the uronic acids present in the initial raw material-between 8% and 14% (Table 4, recovery data). This suggests that between 86% and 92% of the initial uronic acid constituents should be recovered in the residues after extraction. However, as shown in Table 3 and commented above, in the residues after SWE150/10 and SWE150/20 only 32% and 28% of the initial uronic acids were recovered. This is a clear indication that the acidic PSs were destructed and passed in a form that did not allow them to be precipitated by alcohol in the corresponding extract. Considering the data for the PS yield and recovery of uronic acids (Table 3 and Table 4), it may be concluded that 54% and respectively 64% of the uronic acids were in such a form, contained in the extract but not building the corresponding PSs.

The main free sugars identified in the extracts were Glc, Fru, Suc, and Mal. Free sugars remained unchanged at a lower temperature, but with an increase in temperature, even for a short time, their total sum amount was drastically decreased. However, with an increase in the duration of extraction, quantitative changes were observed not only in disaccharides but also in the monosaccharides Glc and Fru, the amounts of which decreased most probably due to thermal destruction. On the other hand, there was an increase in the level of maltose, which is a key structural motif of amylose and maltodextrins. That was an indication of the destruction of glucans of the starch type.

In general, the duration of the treatment at a higher temperature was extremely critical for the recovery of the herbal carbohydrates in the SWE extracts. The enrichment of the extracts with RA and PSs was achieved in SWE100/20 based on their yields. Interestingly, bioactive herbal pectins can be useful for support of immune health, thus their presence in the extracts is positive [64]. The evaluation of the content of free sugars in the SWE extracts is essential from a nutritional point of view. It can evaluate their application in functional and especially in dietary nutrition in case of certain health disorders. However, SWE150/10 was characterized by a higher content of caffeic, neochlorogenic acids, and luteolin (Table 2), as it expressed more potent antioxidant activity in vitro.

## 4. Conclusions

The current study indicates for the first time that SWE is an efficient technology for the recovery of RA from lemon balm (*Melissa officinalis*) leaves, without the use of ethanol or other organic solvents. The temperature of 100 °C was enough for the extraction of RA in comparable amounts as with 50% ethanol. However, further elevation of temperature led to thermal degradation of RA and thus decreased its recovery. In addition, a higher temperature yielded higher amounts of dry extracts, which was due to increased degradation of the constituents of primary cell walls (proteins, pectin, and cellulose). By modification of SWE conditions, dry extracts with different purity and content of RA could be obtained. Thus, it could be concluded that subcritical water enriches the obtained extract with other substances native to the herb, such as proteins, sugars, oligosaccharides, PSs, etc. These extracts could be used in the food industry as food antioxidants, or in the development of food supplements and functional foods. However, further studies should investigate whether these components affect the known biological activities of lemon balm extracts, either positively or negatively.

## Figures and Tables

**Table 1:** Chemical characterization and antioxidant activity of lemon balm leaves.

A. Moisture, %	10.0 ± 0.1
B. Crude protein (N × 6.25), %	18.4 ± 0.5
C. Total lipids, %	2.2 ± 0.1
D. Total carbohydrates, %	22.0 ± 0.5
Glucose (Glc)	1.8 ± 0.1
Fructose (Fru)	1.9 ± 0.2
Sucrose (Suc)	0.02 ± 0.0
Maltose (Mal)	0.3 ± 0.0
Total uronic acids	5.4 ± 0.1
Cellulose	5.3 ± 0.6
E. Ash, %	18.1 ± 0.4
F. Lignin (Klason), %	18.7 ± 0.3
G. Phenolics *, mg/100 g	
Total polyphenols	11,491.1 ± 186.8
Rosmarinic acid	1599.9 ± 45
Neochlorogenic acid	96.6 ± 3.3
Caffeic acid	15.5 ± 1.1
Luteolin	23.8 ± 0.1
H. Antioxidant activity *	
ORAC, µmol TE/g DW	1814.6 ± 51.9
HORAC, µmol GAE/g DW	950.2 ± 15.9

Results are presented as mean values ± SD; * Values are calculated for extract C, obtained by the reference extraction method.

**Table 2:** Content of total and individual phenolics and antioxidant activity of freeze-dried lemon balm extracts, obtained by different extraction methods.

Extract	Total Polyphenols, mg/100 g DW	Caffeic Acid, mg/100 g DW	Neochlorogenic Acid, mg/100 g DW	Luteolin, mg/100 g DW	ORAC, µmol TE/g DW	HORAC, µmol GAE/g DW
SWE100/10	30,933 ^a^ ± 267	60.5 ^b^ ± 0.9	408.3 ^c^ ± 1.1	13.4 ^c^ ± 2.1	3796 ^c^ ± 77	1902 ^b^ ± 95
SWE100/20	30,212 ^a^ ± 622	63.1 ^b^ ± 2.5	412.3 ^c^ ± 25.2	14.1 ^c^ ± 1.3	3784 ^c^ ± 45	1879 ^b^ ± 30
SWE150/10	26,102 ^c^ ± 88	292.6 ^a^ ± 18.2	784.9 ^b^ ± 49.2	92.7 ^b^ ± 2.0	3952 ^b^ ± 50	1877 ^b^ ± 30
SWE150/20	27,972 ^b^ ± 221	173.5 ^b^± 11.1	953.1 ^a^ ± 81.3	112.1 ^a^ ± 9.1	4480 ^a^ ± 91	2207 ^a^ ± 86
TADM	27,426 ^b^ ± 163	47.0 ^c^ ± 3.9	177.7 ^d^ ± 1.2	10.4 ^c^ ± 1.2	4347 ^a^ ± 59	2080 ^ab^ ± 78

Results are presented as mean values ± SD. There are no significant differences among values marked with the same letters (a, b, c, d) within individual groups.

**Table 3:** Yield and chemical characterization of residue after subcritical water extraction (*w*/*w*%).

Constituents	SWE100/10	SWE100/20	SWE150/10	SWE150/20
A. Yield of cell wall material, %	77.5 ^b^ ± 1.0	80.5 ^a^ ± 0.5	79.1 ^a^ ± 1.1	77.2 ^b^ ± 0.7
B. Yield of residue, %	71 ^a^ ± 1	66 ^b^ ± 1	53 ^c^ ± 0	48 ^d^ ± 0
C. Crude protein (N×6.25)	21.3 ^ab^ ± 0.5	21.2 ^b^ ± 0.2	21.8 ^a^ ± 0.1	21.2 ^b^ ± 0.1
Recovery, %	84	77	64	56
D. Total uronic acids	8.9 ^a^ ± 0.2	7.3 ^b^ ± 0.1	3.3 ^c^ ± 0.0	3.2 ^c^ ± 0.1
Recovery, %	116	89	32	28
E. Cellulose	7.9 ^c^ ± 0.6	7.3 ^c^ ± 0.2	9.3 ^a^ ± 0.1	8.7 ^b^ ± 0.1
Recovery, %	105	91	93	79
F. Lignin (Klason)	22.8 ^d^ ± 0.3	28.3 ^c^ ± 0.2	35.3 ^b^ ± 0.3	40.5 ^a^ ± 0.1
Recovery, %	88	99	100	104

Results are presented as mean values ± SD. There are no significant differences among values marked with the same letters (a, b, c, d) within individual groups.

**Table 4:** Polysaccharide content and sugar composition of lemon balm subcritical water extracts (*w*/*w*%).

Constituents	SWE100/10	SWE100/20	SWE150/10	SWE150/20
Polysaccharide, % * (g/100 mL extract)	2.0 ^d^ ± 0.0 (0.10)	2.6 ^c^ ± 0.1 (0.13)	3.2 ^b^ ± 0.1 (0.16)	3.6 ^a^ ± 0.1 (0.18)
Total carbohydrates of PS	31 ^d^ ± 0.5	37 ^c^ ± 0.3	66 ^a^ ± 0.8	49 ^b^ ± 0.1
Total uronic acids of PS	26 ^b^ ± 0.2	29 ^a^ ± 0.2	21 ^c^ ± 0.0	13 ^d^ ± 0.0
Recovery, % (100-%)	9.8 (90.2)	14.1 (85.9)	12.6 (87.4)	8.6 (91.4)
Glucose (Glc)	1.5 ^a^ ± 0.1	1.5 ^a^ ± 0.1	1.7 ^a^ ± 0.2	0.9 ^b^ ± 0.1
Fructose (Fru)	1.9 ^a^ ± 0.0	1.9 ^a^ ± 0.2	1.8 ^a^ ± 0.1	1.0 ^b^ ± 0.1
Sucrose (Suc)	1.0 ^a^ ± 0.1	1.1 ^a^ ± 0.0	0.8 ± ^b^ 0.0	0.2 ^c^ ± 0.0
Maltose (Mal)	0.2 ^b^ ± 0.0	0.1 ^c^ ± 0.0	0.2 ^b^ ± 0.0	0.4 ^a^ ± 0.0
Total	4.7	4.6	4.5	2.5

* calculated as a percent of initial plant material; Results are presented as mean values ± SD. There are no significant differences among values marked with the same letters (a, b, c, d) within individual groups.

## Data Availability

Data are contained within the article and Supplementary Materials.

## Supplementary Materials

The following supporting information can be downloaded at: https://www.mdpi.com/article/10.3390/antiox12040888/s1; Supplementary Figure S1. Chromatograms from: (A) HPLC determination of rosmarinic acid (λ = 330 nm) in accordance to the pharmacopoeia method described in Section 2.6; RT of rosmarinic acid—10.6 min: (B) HPLC determination of phenolic compounds (λ = 280 nm) according to Section 2.7; RT: neochlorogenic acid—2.1 min; caffeic acid—4.8 min; rosmarinic acid—21.2 min; luteolin—31.3 min. Supplementary Figure S2. Recovery of (A) Total polyphenols and (B) Rosmarinic acid from lemon balm dry leaves during continuous static maceration. Results are presented as mean values ± standard deviation (SD). There are no significant differences among values marked with the same letters within individual groups.

## References

[1] Shekarchi M., Hajimehdipoor H., Saeidnia S., Gohari A.R., Hamedani M.P. (2012). Comparative study of rosmarinic acid content in some plants of *Labiatae* family. Pharmacogn. Mag..

[2] Kintzios S., Nikolaou A., Skoula M. (1999). Somatic embryogenesis and in vitro rosmarinic acid accumulation in *Salvia officinalis* and *S. fruticosa* leaf callus cultures. Plant Cell Rep..

[3] Petersen M. (2013). Rosmarinic acid: New aspects. Phytochem. Rev..

[4] Lagouri V., Nisteropoulou E. (2009). Antioxidant properties of *O. onites*, *T. vulgaris* and *O. basilicum* species grown in Greece and their total phenol and rosmarinic acid content. J. Food Lipids.

[5] Cao H., Cheng W.X., Li C., Pan X.L., Xie X.G., Li T.H. (2005). DFT study on the antioxidant activity of rosmarinic acid. J. Mol. Struct. Theochem..

[6] Amit S.K., Uddin M.M., Rahman R., Islam S.M.R., Khan M.S. (2017). A review on mechanisms and commercial aspects of food preservation and processing. Agric. Food Secur..

[7] Vasileva I., Denkova R., Chochkov R., Teneva D., Denkova Z., Dessev T., Denev P., Slavov A. (2018). Effect of lavender (*Lavandula angustifolia*) and melissa (*Melissa officinalis*) waste on quality and shelf life of bread. Food Chem..

[8] Farahmandfar R., Naeli M.H., Naderi M., Asnaashari M. (2019). Stabilizing corn oil using the lemon balm (*Melissa officinalis*) antioxidants extracted by subcritical water. J. Food Sci. Technol..

[9] Vara S., Karnena M.K., Dwarapureddi B.K., Grumezescu A.M., Holban A.M. (2019). Natural Preservatives for Nonalcoholic Beverages. Preservatives and Preservation Approaches in Beverages.

[10] Caleja C., Barros L., Barreira J.C.M., Ciric A., Sokovic M., Calhelha R.C., Beatrizb M., Oliveira P.P., Ferreira I.C.F.R. (2018). Suitability of lemon balm (*Melissa officinalis* L.) extract rich in rosmarinic acid as a potential enhancer of functional properties in cupcakes. Food Chem..

[11] Klisurova D., Petrova I., Ognyanov M., Georgiev Y., Kratchanova M., Denev P. (2019). Co-pigmentation of black chokeberry (*Aronia melanocarpa*) anthocyanins with phenolic co-pigments and herbal extracts. Food Chem..

[12] Carocho M., Morales P., Ferreira I.C.F.R. (2015). Natural food additives: Quo vadis?. Trends Food Sci. Technol..

[13] Costa P., Sarmento B., Gonçalves S., Romano A. (2013). Protective effects of *Lavandula viridis* L’her extracts and rosmarinic acid against H_2_O_2_-induced oxidative damage in A172 human astrocyte cell line. Ind. Crops Prod..

[14] Wang S.J., Chen Q., Liu M.Y., Yu H.Y., Xu J.Q., Wu J.Q., Zhang Y., Wang T. (2019). Regulation effects of rosemary (*Rosmarinus officinalis* Linn.) on hepatic lipid metabolism in OA induced NAFLD rats. Food Funct..

[15] Georgiev M., Pastore S., Lulli D., Alipieva K., Kostyuk V. (2012). Verbascum xanthophoeniceum-derived phenylethanoid glycosides are potent inhibitors of inflammatory chemokines in dormant and interferon-gamma-stimulated human keratinocytes. J. Ethnopharmacol..

[16] Zhao L., Zhang Y., Liu G., Hao S., Wang C., Wang Y. (2018). Black rice anthocyanin-rich extract and rosmarinic acid, alone and in combination, protect against DSS-induced colitis in mice. Food Funct..

[17] Topal M., Gulcin I. (2022). Evaluation of the in vitro antioxidant, antidiabetic and anticholinergic properties of rosmarinic acid from rosemary (*Rosmarinus officinalis* L.). Biocatal. Agric. Biotechnol..

[18] Vasileva L.V., Savova M.S., Tews D., Wabitsch M., Georgiev M.I. (2021). Rosmarinic acid attenuates obesity and obesity-related inflammation in human adipocytes. Food Chem. Toxicol..

[19] Elufioyea T.O., Habtemariamb S. (2019). Hepatoprotective effects of rosmarinic acid: Insight into its mechanisms of action. Biomed. Pharmacother..

[20] Luo C., Zou L., Sun H., Peng J., Gao C., Bao L., Ji R., Jin Y., Sun S. (2020). A Review of the anti-inflammatory effects of rosmarinic acid on inflammatory diseases. Front. Pharmacol..

[21] Stansbury J. (2014). Rosmarinic acid as a novel agent in the treatment of allergies and asthma. J. Restor. Med..

[22] Noguchi-Shinohara M., Ono K., Hamaguchi T., Iwasa K., Nagai T., Kobayashi S., Nakamura H., Yamada M. (2015). Pharmacokinetics, safety and tolerability of *Melissa officinalis* extract which contained rosmarinic acid in healthy individuals: A randomized controlled trial. PLoS ONE.

[23] Verlag G.T. (2003). The Scientific Foundation for Herbal Medicinal Products.

[24] Hänsel R., Keller K., Rimpler H., Schneider G. (1993). Hagers Handbuch.

[25] Zhao J., Xu L., Jin D., Xin Y., Tian L., Wang T., Zhao D., Wang Z., Wang J. (2022). Rosmarinic acid and related dietary supplements: Potential applications in the prevention and treatment of cancer. Biomolecules.

[26] Kwon Y.O., Hong J.T., Oh K.W. (2017). Rosmarinic acid potentiates pentobarbital-induced sleep behaviors and non-rapid eye movement (NREM) sleep through the activation of GABA_A_-ergic systems. Biomol. Ther..

[27] European Medicine Agency, Committee on Herbal Medicinal Products (HMPC) (2013). Community Herbal Monograph on *Melissa officinalis* L., Folium, 196745/2012. https://www.ema.europa.eu/en/documents/herbal-monograph/final-community-herbal-monograph-melissa-officinalis-l-folium_en.pdf.

[28] (2013). Melissa Leaf. Ph Eur Monograph 1447, European Pharmacopoeia.

[29] (2013). Melissa Leaf Dry Extract. Ph Eur Monograph 2524, European Pharmacopoeia.

[30] Marchev A.S., Vasileva L.V., Amirova K.M., Savova M.S., Koycheva I.K., Balcheva-Sivenova Z.P., Vasileva S.M., Georgiev M.I. (2021). Rosmarinic acid—From bench to valuable applications in food industry. Trends Food Sci. Technol..

[31] Iwaia M., Ohta M., Tsuchiyac H., Suzukia T. (2010). Enhanced accumulation of caffeic acid, rosmarinic acid and luteolin-glucoside in red perilla cultivated under red diode laser and blue LED illumination followed by UV-A irradiation. J. Funct. Foods.

[32] Hadi N., Sefidkon F., Shojaeiyan A., Šiler B., Jafari A.A. (2017). Phenolics’ composition in four endemic *Nepeta* species from Iran cultivated under experimental field conditions: The possibility of the exploitation of *Nepeta* germplasm. Ind. Crops Prod..

[33] Nourozi E., Hosseini B., Maleki R., Mandoulakani B. (2019). Pharmaceutical important phenolic compounds overproduction and gene expression analysis in *Dracocephalum kotschyi* hairy roots elicited by SiO_2_ nanoparticle. Ind. Crops Prod..

[34] Chen S.Y., Wang G.Y., Lin J.H., Yen G.C. (2020). Antioxidant and anti-inflammatory activities and bioactive compounds of the leaves of *Trichodesma khasianum* Clarke. Ind. Crops Prod..

[35] Mabrouki H., Duarte C.M.M., Akretche D.E. (2018). Estimation of total phenolic contents and in vitro antioxidant and antimicrobial activities of various solvent extracts of *Melissa officinalis* L.. Arab. J. Sci. Eng..

[36] Khalaja A., Khanib S. (2018). Spasmolytic effects of hydro-alcoholic extract of *Melissa officinalis* on isolated rat ileum. J. Rep. Pharm. Sci..

[37] Hong E., Kim G. (2010). Comparison of extraction conditions for phenolic, flavonoid content and determination of rosmarinic acid from *Perilla frutescens* var. acuta. Int. J. Food Sci..

[38] Ngoa Y.L., Laua C.H., Chua L.S. (2018). Review on rosmarinic acid extraction, fractionation and its anti-diabetic potential. Food Chem. Toxicol..

[39] Mirona T.L., Herrerob M.E., Nezb I. (2013). Enrichment of antioxidant compounds from lemon balm (*Melissa officinalis*) by pressurized liquid extraction and enzyme-assisted extraction. J. Chromatogr. A.

[40] Quintana S., Villanueva-Bermejo D., Reglero G., García-Risco M., Fornari T. (2019). Supercritical antisolvent particle precipitation and fractionation of rosemary (*Rosmarinus officinalis* L.) extracts. J. CO2 Util..

[41] Hirondart M., Rombaut N., Fabiano-Tixier A.S., Bily A., Chemat F. (2020). Comparison between pressurized liquid extraction and conventional soxhlet extraction for rosemary antioxidants, yield, composition, and environmental footprint. Foods.

[42] Liu T., Sui X., Zhang R., Yang L., Zu Y., Zhang L., Zhang Y., Zhang Z. (2011). Application of ionic liquids based microwave-assisted simultaneous extraction of carnosic acid, rosmarinic acid and essential oil from *Rosmarinus officinalis*. J. Chromatogr. A.

[43] Saad E., El Gohary N., Abdel-Halim M., Handoussa H., El Nashar R., Mizaikoff B. (2021). Molecularly imprinted polymers for selective extraction of rosmarinic acid from *Rosmarinus officinalis* L.. Food Chem..

[44] Cheng Y., Xue F., Yu S., Du S., Yang Y. (2021). Subcritical water extraction of natural products. Molecules.

[45] Plaza M., Turner C. (2015). Pressurized hot water extraction of bioactives. TrAC Trends Anal. Chem..

[46] (2016). Determination of Protein in Foods. National Food Safety Standard (NFSS) of the People’s Republic of China.

[47] DuBois M., Gilles K.A., Hamilton J.K., Rebers P.A., Smith F. (1956). Colorimetric method for determination of sugars and related substances. Anal. Chem..

[48] Nenov N., Govedarov M., Baldjieva T. (2022). Pressurized hot water system for green extraction of valuable compounds from biomass and its performance. J. Balkan Ecol..

[49] Singleton V., Rossi J. (1965). Colorimetry of total phenolic with phosphomolibdiphosphotungstic acid reagents. Am. J. Enol. Vitic..

[50] Ou B., Hampsch-Woodill M., Prior R.L. (2001). Development and validation of an improved oxygen radical absorbance capacity assay using fluorescein as the fluorescence probe. J. Agric. Food Chem..

[51] Denev P., Ciz M., Ambrozova G., Lojek A., Yanakieva I., Kratchanova M. (2010). Solid-phase extraction of berries’ anthocyanins and evaluation of their antioxidative properties. Food Chem..

[52] Ou B., Hampsch-Woodill M., Flanagan J., Deemer E.K., Prior R.L., Huang D. (2002). Novel fluorometric assay for hydroxyl radical prevention capacity using fluorescein as the probe. J. Agric. Food Chem..

[53] Updegraff D.M. (1969). Semimicro determination of cellulose in biological materials. Anal. Biochem..

[54] Dence C.W., Lin S.Y., Dence C.W. (1992). The determination of lignin. Methods in Lignin Chemistry.

[55] Fukushima R.S., Kerley M.S., Ramos M.H., Porter J.H., Kallenbach R.L. (2015). Comparison of acetyl bromide lignin with acid detergent lignin and Klason lignin and correlation with in vitro forage degradability. Anim. Feed Sci. Technol..

[56] Putnik P., Kovačević D.B., Penić M., Fegeš M., Dragović-Uzelac V. (2016). Microwave-assisted extraction (MAE) of dalmatian sage leaves for the optimal yield of polyphenols: HPLC-DAD identification and quantification. Food Anal. Methods.

[57] Caleja C., Barros L., Prieto M.A., Barreiro M.F., Oliveira M.B.P.P., Ferreira I.C.F.R. (2017). Extraction of rosmarinic acid from *Melissa officinalis* L. by heat-, microwave- and ultrasound-assisted extraction techniques: A comparative study through response surface analysis. Sep. Purif. Technol..

[58] Zhang Y., Smuts J.P., Dodbiba E., Rangarajan R., Lang J.C., Armstrong D.W. (2012). Degradation study of carnosic acid, carnosol, rosmarinic acid, and rosemary extract (*Rosmarinus officinalis* L.) assessed using HPLC. J. Agric. Food Chem..

[59] Jeong G.H., Cho J.H., Jo C., Lee S., Lee S.S., Bai H.W., Chung B.Y., Kim T.H. (2018). Gamma irradiation-assisted degradation of rosmarinic acid and evaluation of structures and anti-adipogenic properties. Food Chem..

[60] Ferraro V., Madureira A., Sarmento B., Gomes A., Pintado M. (2015). Study of the interactions between rosmarinic acid and bovine milk whey protein *α*-lactalbumin, *β*-lactoglobulin and lactoferrin. Food Res. Int..

[61] Li Y., Qi H., Fan M., Zhu Z., Zhan S., Li L. (2020). Quantifying the efficiency of *o*-benzoquinones reaction with amino acids and related nucleophiles by cyclic voltammetry. Food Chem..

[62] Patora J., Klimek B. (2002). Flavonoids from lemon balm (*Melissa officinalis* L., *Lamiaceae*). Acta Pol. Pharm..

[63] Barros L., Dueñas M., Dias M.I., Sousa M.J., Santos-Buelga C., Ferreira I.C.F.R. (2013). Phenolic profiles of cultivated, in vitro cultured and commercial samples of *Melissa officinalis* L. infusions. Food Chem..

[64] Georgiev Y.N., Paulsen B.S., Kiyohara H., Ciz M., Ognyanov M.H., Vasicek O., Rise F., Yamada H., Denev P.N., Lojek A. (2017). The common lavender (*Lavandula angustifolia* Mill.) pectic polysaccharides modulate phagocytic leukocytes and intestinal Peyer’s patch cells. Carbohydr. Polym..

