# Supplementary material for: Subcritical Water Extraction of Rosmarinic Acid from Lemon Balm (Melissa officinalis L.) and Its Effect on Plant Cell Wall Constituents
Source: Antioxidants (Basel). 2023 Apr 5;12(4):888. doi: 10.3390/antiox12040888 (PMC10135751; doi:10.3390/antiox12040888)
Supplement: Supplementary file 1 [file antioxidants-12-00888-s001.zip › antioxidants-2283817-supplementary.pdf]

# Supplementary Materials

# Subcritical Water Extraction of Rosmarinic Acid from Lemon Balm (*Melissa officinalis* L.) and its Effect on Plant Cell Wall Constituents

**Ana Atanasova, Ani Petrova, Desislava Teneva, Manol Ognyanov, Yordan Georgiev, Nenko Nenov, Petko Denev**

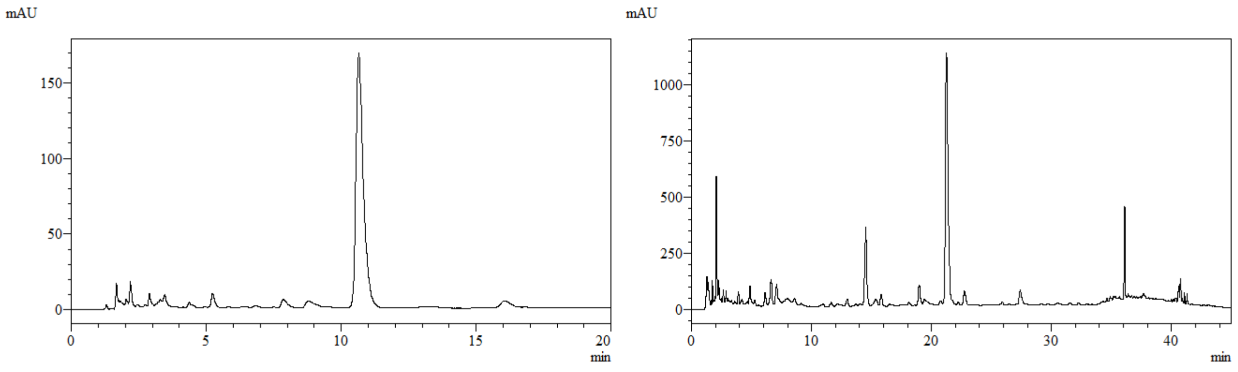


A B

Supplementary Figure S1. Chromatograms from: A) HPLC determination of rosmarinic acid (λ = 330 nm) in accordance to the pharmacopoeia method described in section 2.6; RT of rosmarinic acid - 10.6 min: B) HPLC determination of phenolic compounds (λ = 280 nm) according to section 2.7.; RT: neochlorogenic acid – 2.1 min; caffeic acid - 4.8 min; rosmarinic acid – 21.2 min; luteolin – 31.3 min;

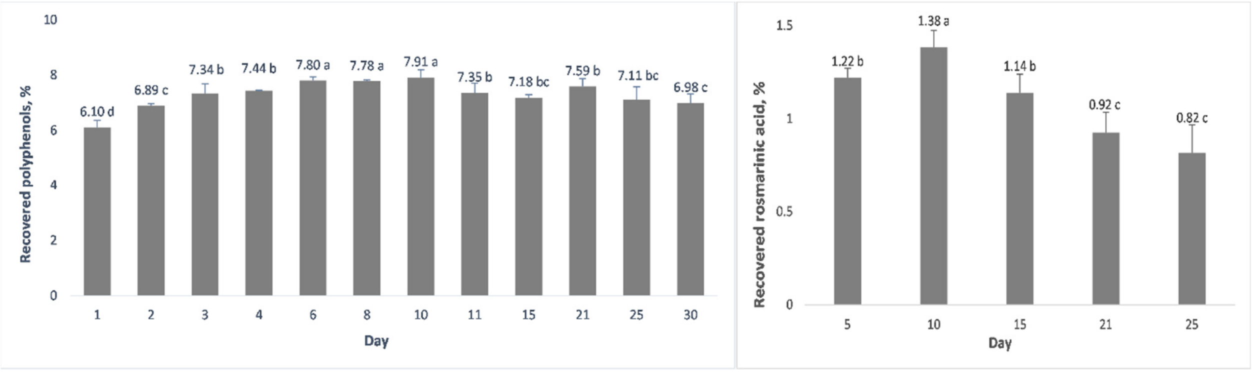


A B

Supplementary Figure S2. Recovery of A) Total polyphenols and B) Rosmarinic acid from lemon balm dry leaves during continuous static maceration. Results are presented as mean values ± standard deviation (SD). There are no significant differences among values marked with the same letters within individual groups.
